# *POT1* mutations are frequent and associated with Ki-67 index in canine diffuse large B-cell lymphoma

**DOI:** 10.3389/fvets.2022.968807

**Published:** 2022-08-09

**Authors:** Antonella Fanelli, Laura Marconato, Luca Licenziato, Lucia Minoli, Nicole Rouquet, Luca Aresu

**Affiliations:** ^1^Department of Veterinary Sciences, University of Turin, Turin, Italy; ^2^Department of Veterinary Medical Sciences, University of Bologna, Bologna, Italy; ^3^Hastim, Toulouse, France

**Keywords:** diffuse large B-cell lymphoma, dog, *POT1*, mutation, Ki-67

## Abstract

Diffuse large B-cell lymphoma (DLBCL) represents one of the most frequent and deadliest neoplasia in dogs worldwide and is characterized by a remarkable degree of clinical heterogeneity, with poor chances to anticipate the outcome. Even if in the last years some recurrently mutated genes have been identified, the genetic origin of canine DLBCL (cDLBCL) is not yet completely understood. The aim of the present study was to assess the prevalence of *POT1* mutations in cDLBCL and to elucidate the role of such gene in the pathogenesis of this tumor. Mutations in *POT1* were retrieved in 34% of cases, in line with previous reports, but no significant associations with any clinico-pathological variable were identified. Likewise, *POT1* mutations are not predictive of worse prognosis. Interestingly, Ki-67 index was significantly higher in dogs harboring *POT1* mutations compared to wild-type ones. These results suggest that *POT1* mutations may exert their pathogenic role in cDLBCL by promoting cellular proliferation.

## Introduction

Non-Hodgkin lymphomas (NHLs) represent the most common hematological malignancies in dogs, and diffuse large B-cell lymphoma (DLBCL) is the most frequent histotype accounting for 50–60% of cases (1). Canine DLBCL (cDLBCL) is characterized by an aggressive behavior, being associated with a poor prognosis. Although the majority of dogs undergoing treatment achieves complete responses, these are short-lasting. Moreover, treatment response may be heterogeneous and difficult to predict, suggesting the existence of different molecular subtypes not identifiable through the routinely performed diagnostic tests (2).

Within this context, the molecular pathogenesis of cDLBCL is not yet completely understood. Gene expression profiling (GEP) and RNA sequencing studies unraveled the existence of two distinct subgroups resembling human activated B cell—like (ABC) DLBCL and germinal center B cell—like (GCB) DLBCL, showing significantly different survival times (3, 4). Recurrently mutated genes (*TRAF3, SETD2, POT1, TP53, FBXW7*) were also identified through whole exome sequencing (WES) experiments (5–7); however, the incomplete clinical data in most of the studies did not allow to properly attribute a significance to these genetic variants. Through the integration of “omics” data and clinico-pathological features, we recently developed a predictive model that allowed to identify some negative prognostic factors, including *TP53* mutational status, that are able to define a subgroup of dogs not gaining significant improvement from the addition of immunotherapy to the standard CHOP-based chemotherapy (https://compbiomed.hpc4ai.unito.it/canine-dlbcl/) (7).

POT1 (Protection of Telomeres 1) is one of the six telomere-specific binding proteins constituting the shelterin complex. This complex recognizes TTAGGG repeats and is involved in telomere length regulation and maintenance (8). Among shelterin proteins, POT1 directly binds single-stranded (ss) G-rich 3′ telomere overhangs, regulates telomerase activity and protects chromosome ends from being recognized as double strand breaks by DNA damage repair (DDR) mechanisms (9–11). Indeed, POT1 loss has been shown to be implicated in telomere lengthening and increased chromosomal instability, thus promoting tumorigenesis (12). In humans, germline mutations in *POT1* have been identified in familial cases of melanoma (13–15), glioma (16), colorectal cancer (17), angiosarcoma (18), Hodgkin lymphoma (19) and chronic lymphocytic leukemia (CLL) (20). Furthermore, somatic mutations have been retrieved in many cancer types (21), including CLL (22, 23), in which *POT1* mutations were associated with reduced overall survival in patients treated with chemo-immunotherapy based on chlorambucil-rituximab or chlorambucil-obinutuzumab (24).

In dogs, the role of *POT1* aberrations in tumorigenesis and prognosis is largely unexplored. One study assessed the prevalence of *TRAF3* and *POT1* mutations in a cohort of canine B-cell lymphomas (cBCL), but their clinical significance was not investigated (25). We previously reported *POT1* mutations in 29% of 77 cDLBCLs analyzed through WES. Furthermore, we also retrieved an association between *POT1* mutations and reduced lymphoma specific survival (LSS) in dogs treated with chemo-immunotherapy (7).

The aim of the present study was to extend *POT1* genetic analysis to an independent cohort of dogs with DLBCL treated with chemo-immunotherapy in order to further investigate the prognostic significance and the role of such gene in the pathogenesis of this tumor.

## Materials and methods

### Animals

Fifty-six cDLBCLs were retrieved from the archive of the Canine Lymphoma Biobank (26). To be enrolled in this study, dogs had to be treated with chemo-immunotherapy consisting of the administration of APAVAC vaccine in addition to a CHOP-based standardized protocol as previously described (27). For all dogs, signalment and the following clinico-pathological features were available: clinical stage, substage, immunophenotype assessed by flow cytometry (FC) on lymph node (LN), peripheral blood (PB) and bone marrow (BM) infiltration, serum lactate dehydrogenase (LDH) level and whether dogs had been pre-treated with steroids. All dogs underwent lymphadenectomy for routine histologic analysis and immunohistochemistry (IHC) (CD3 and CD20), vaccine preparation and DNA extraction. Whole blood was also collected in EDTA tubes to provide matched-normal tissue.

Time to progression (TTP), lymphoma-specific survival (LSS) and cause of death were also available. TTP was measured as the intervening time between the start of treatment and disease progression. LSS was calculated from the start of treatment to death from lymphoma.

The study did not fall within the application areas of the Italian Legislative Decree 26/2014 which governs the protection of animals used for scientific or educational purposes; therefore, ethical approval was waived for this study. All dogs enrolled received the current gold-standard treatment. Owners gave their written informed consent.

### DNA extraction and quantification

DNA was extracted from fresh frozen tumor tissue and whole blood using DNeasy Blood & Tissue kit (Qiagen, Hilden, Germany) following manufacturer's instructions. DNA quality and quantity were assessed using NanoDrop 2000 Spectrophotometer (ThermoFisher, Waltham, MA, USA).

### Primer design and PCR amplification

Primers to specifically amplify the whole coding region, 3′- and 5′- untranslated regions (UTRs) and the intron-exon junctions of *POT1* gene were designed using Primer3 (https://primer3.ut.ee/) and Primer-BLAST (https://www.ncbi.nlm.nih.gov/tools/primer-blast/) tools (Supplementary Table 1). PCR was performed using GoTaq^®^ G2 Flexi DNA Polymerase kit (Promega, Madison, WI, USA) with 50 ng of gDNA in a final volume of 15 μl. Cycling conditions were the following: initial denaturation at 95°C for 5 min, 35 cycles at 95°C for 30 s, at 60°C for 30 s and at 72°C for 1 min, and final extension at 72°C for 10 min. Exon 18 was amplified using a Touchdown PCR protocol (28). Briefly, the touchdown phase was conducted with 20 cycles at annealing temperature of 65°C decreasing 0.5°C at each cycle, while the second phase was conducted with 25 cycles at annealing temperature of 55°C. PCR products were visualized on 1.5% agarose gel (Bio-rad, Hercules, CA, USA) and purified using the ExoSAP-IT PCR Product Cleanup Reagent (Applied Biosystems, Foster City, CA, USA).

### Sequencing analysis

Purified PCR products were sequenced in-house in the forward or reverse direction using the BigDye Terminator v1.1 Cycle Sequencing Kit (Applied Biosystems) following manufacturer's instructions and analyzed on a SeqStudio Genetic Analyzer (Applied Biosystems). Sequencing electropherograms were manually inspected using Chromas v2.6.6 software. Mutations were identified by comparing sequences obtained from each tumor to the ROS Cfam 1.0 reference genome and were classified as somatic when absent in matched-normal tissue. Mutations consequences were assessed using Variant Effect Predictor (VEP) tool from Ensembl (https://www.ensembl.org/Tools/VEP) referring to ENSCAFT00845010067.1 Ensembl canonical transcript.

### Ki-67 evaluation

Immunohistochemical analysis was performed on serial sections of formalin-fixed paraffin blocks. Antigen retrieval was performed in Tris-EDTA buffer (pH 9.0) and a mouse monoclonal (MIB-1) anti-Ki-67 primary antibody was employed (Dako, Glostrup, Denmark) with a final dilution of 1:500. Detection was performed using the avidin-biotin peroxidase complex technique with the Vectastain Elite ABC Kit (Vector Laboratories, Burlingame, CA, USA) and slides were blindly reviewed by two authors (L.A., L.Mi.). The Ki-67 index was determined by counting the number of positive cells per 1,000 randomly selected cells excluding necrotic areas, as previously described (29).

### Statistical analyses

All statistical analyses were conducted in R environment. Continuous variables were tested for normal distribution by means of a Shapiro-Wilk test. Differences between *POT1* mutated and wild-type dogs regarding age, weight, bone marrow and peripheral blood infiltration were assessed by means of a Student *t*-test for normally distributed variables, otherwise by Mann-Whitney test. For categorical variables, 2x2 contingency tables were prepared and Fisher's exact test was conducted to assess possible associations with *POT1* mutational status. Differences in Ki-67 index across breed (pure vs. mixed), sex (female vs. male), age (<9 years vs. ≥9 years), weight (<26.3 kg vs. ≥26.3 kg), substage (a vs. b), BM infiltration (yes vs. no), LDH activity (normal vs. increased), pretreatment with steroids (yes vs. no) and *POT1* mutational status (mutated, mut vs. wild-type, wt) groups were assessed by Mann-Whitney test. Differences in Ki-67 index across groups stratified based on stage were evaluated using a Kruskal-Wallis test. Possible correlations between Ki-67 index and age, weight, BM and PB infiltration (%) were assessed using a Spearman correlation test. Benjamini-Hochberg correction was applied to multiple testing. Survival analysis was conducted using *survival* and *survminer* R packages. All the previously described clinico-pathological variables, *POT1* mutational status and Ki-67 index were tested for their influence on both TTP and LSS by means of univariate and multivariate Cox proportional-hazards model. Dogs lost to follow-up or dead for lymphoma unrelated causes before disease progression were censored for TTP analysis. Dogs dead from lymphoma unrelated causes were censored for LSS analysis. Multivariate analysis was conducted using variables with a *P* < 0.200. For categorical variables, survival curves were constructed using the Kaplan-Meier method and compared by means of log-rank test. For all statistical analyses, significance threshold was set at *P* < 0.05.

## Results

### Subjects

Fifty-six dogs with histologically confirmed DLBCL met the inclusion criteria. Complete signalment and clinico-pathological features are reported in Supplementary Table 2. Mixed-breed dogs (*n* = 18, 32%), Rottweiler, Dobermann, and Golden Retriever dogs (*n* = 4 each, 7%) represented the most prevalent breeds. Twenty-nine (52%) dogs were females and 27 (48%) were males. Median age at diagnosis was 9 years (range: 3–15) while median weight was 26.3 kg (range: 6.9–54.9). Regarding clinical stage, only two (4%) dogs had stage III disease, 13 (23%) had stage IV and 41 (73%) had stage V. At diagnosis, 38 (68%) dogs were asymptomatic (substage a), while 18 (32%) showed symptoms (substage b). Twenty-three (41%) dogs presented BM infiltration (>3% of neoplastic cells) and 29 (52%) had PB infiltration (>3% of neoplastic cells). Both TTP and LSS were significantly shorter in dogs receiving corticosteroids before starting chemo-immunotherapy (*P* = 0.01 and *P* = 0.03, respectively). However, this association was not confirmed at multivariate analysis (Supplementary Table 3).

### *POT1* mutational analysis

**Figure 1:**
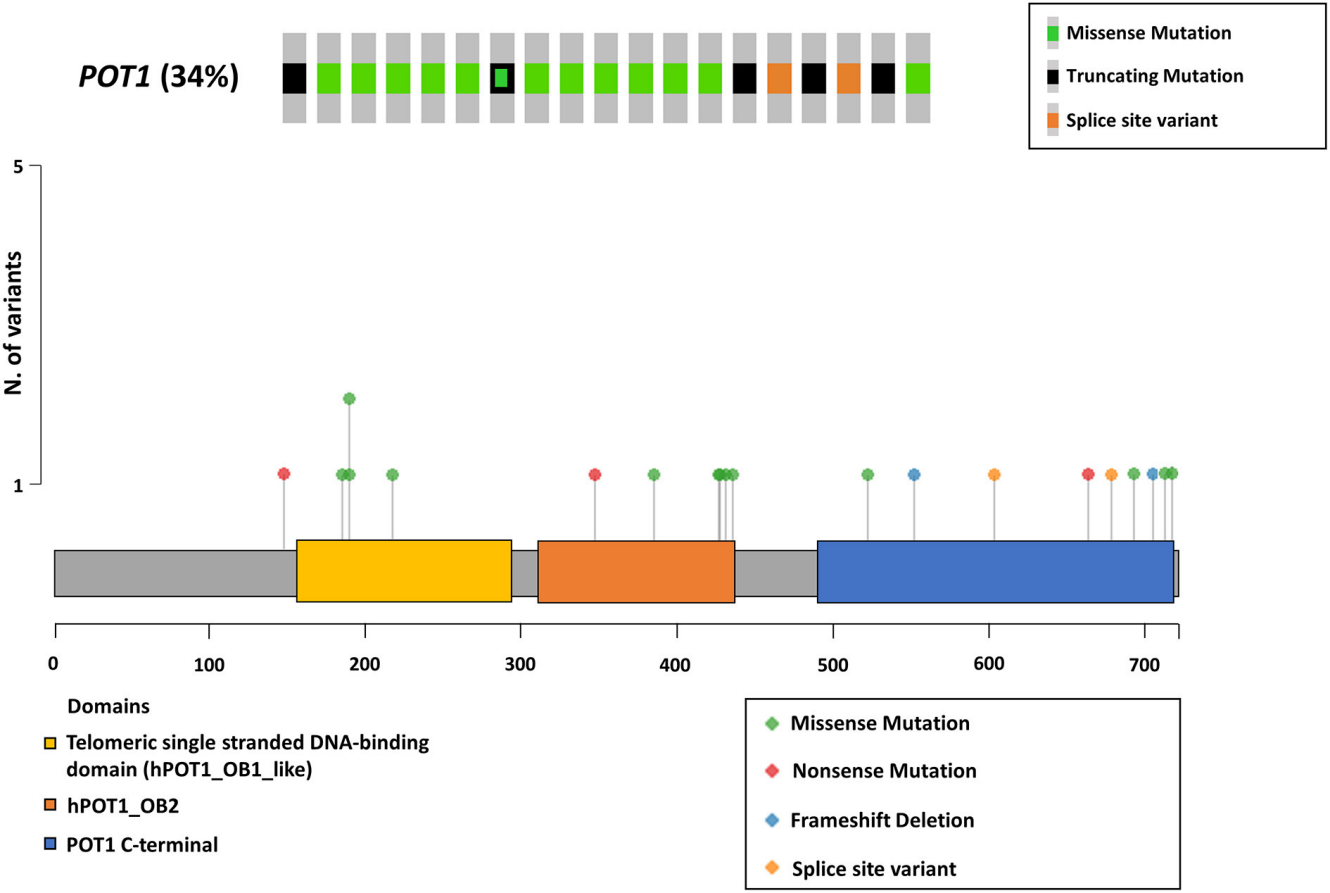
*POT1* somatic mutations. The upper panel shows dogs harboring a protein coding somatic mutation in *POT1* gene. Frameshift and non-sense variants are both indicated as truncating mutations. The Oncoprint was generated using the *Oncoprinter* tool from cBioPortal (https://www.cbioportal.org/oncoprinter). The lower panel shows the lollipop plot of mutations retrieved in the study cohort. The height of each bar represents the frequency at which each mutation occurred. Functional protein domains are also depicted. The lollipop plot was generated using the *maftools* R package and modified to adapt to canine protein (F1P716_CANLF from UniProt).

**Figure 2:**
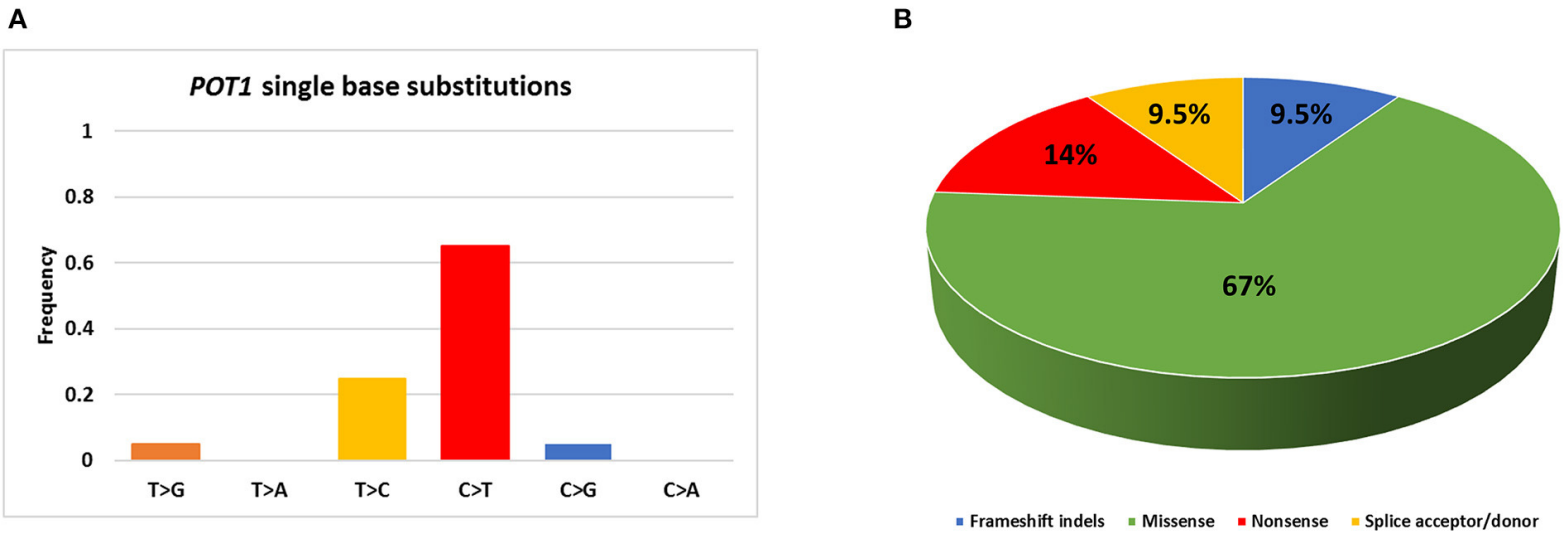
Summary of *POT1* mutations identified in the present study. **(A)** Percentage of single base substitutions; **(B)** Percentage of mutations belonging to each class.

The whole coding sequence, 3′- and 5′ UTRs and intron-exon junctions of *POT1* gene were Sanger sequenced. It was not possible to sequence exon 10 for case #28 and this sample was excluded from further statistical analyses. The complete list of *POT1* mutations is reported in Table 1. Twenty different non-synonymous variants were retrieved in 19/56 (34%) dogs (Figure 1). C>T transitions represented the most frequent single nucleotide substitution (65%) (Figure 2A). In total, we identified 13 different missense variants, all predicted as deleterious by Sorting Intolerant from Tolerant (SIFT) tool, non-sense mutations (*n* = 3), splice donor/acceptor variants (*n* = 2), and two frameshift deletions (Figure 2B). Four variants were already reported in dbSNP and five variants were previously identified in canine B cell lymphoma sequencing studies (6, 7) (Table 1). Three dogs harbored a mutation affecting codon 187 (p.G187R; p.G187E), while two dogs presented two different aberrations (case #1 and case #47). Case #1 presented a non-sense (p.W331X) and a missense (p.L689P) variant in exons 10 and 18, respectively. Case #47 harbored two missense variants (p.E755K and p.C756Y) both in exon 20. In both cases it was not possible to establish if mutations were biallelic or occurred on the same allele. In two dogs (case #9 and case #47), mutations identified in tumor were also retrieved in DNA extracted from whole blood. Nevertheless, we classified mutations as somatic rather than germline since these dogs showed a high percentage of PB infiltration (25% in case #9 and 32% in case #47) that may have determined a mutant allele fraction detectable from Sanger sequencing (sensitivity of 15–20%). Finally, we also retrieved four different 5′UTR variants in 7 dogs that are not reported in public databases (Table 1). However, since the analysis of the matched-normal tissue revealed a germline origin, such variants were not considered for downstream statistical analyses.

**Table 1:** *POT1* variants identified in 56 cDLBCL.

**Case ID#**	**Exon**	**Consequence**	**CDS**	**Protein**	**SIFT**	**Origin**	**dbSNP**	**Reported in cBCL**
40	1	5′UTR	c.-210 G>A	-	-	Germline	-	-
40	1	5′UTR	c.-209 A>C	-	-	Germline	-	-
22	1	5′UTR	c.-141insG	-	-	Germline	-	-
50	1	5′UTR	c.-141insG	-	-	Germline	-	-
39	1	5′UTR	c.-141insG	-	-	Germline	-	-
35	1	5′UTR	c.-141insG	-	-	Germline	-	-
15	1	5′UTR	c.-135 C>T	-	-	Germline	-	-
25	1	5′UTR	c.-135 C>T	-	-	Germline	-	-
44	6	Non-sense	c.C440G	p.S147X	-	Somatic	-	-
55	7	Missense	c.C545T	p.P182L	Deleterious	Somatic	-	-
10	7	Missense	c.G559A	p.G187R	Deleterious	Somatic	-	-
21	7	Missense	c.G560A	p.G187E	Deleterious	Somatic	-	-
50	7	Missense	c.G560A	p.G187E	Deleterious	Somatic	-	-
36	8	Missense	c.G680A	p.R227H	Deleterious	Somatic	rs850707945	Elvers (6)
1	10	Non-sense	c.G993A	p.W331X	-	Somatic	-	Giannuzzi (7)
11	10	Missense	c.A1114G	p.N372D	Deleterious	Somatic	rs851843386	Elvers (6)
49	11	Missense	c.G1246A	p.G416S	Deleterious	Somatic	-	-
5	11	Missense	c.C1250T	p.T417I	Deleterious	Somatic	-	-
25	11	Missense	c.G1262A	p.R421Q	Deleterious	Somatic	rs851542014	Elvers (6); Giannuzzi (7)
31	11	Missense	c.T1277C	p.L426S	Deleterious	Somatic	-	-
32	14	Missense	c.A1559C	p.Y520S	Deleterious	Somatic	-	-
15	15	Frameshift deletion	c.1655delT	p.L552X	-	Somatic	-	-
9	15	Splice donor	-	-	-	Somatic	-	-
35	17	Non-sense	c.G2024A	p.W675X	-	Somatic	rs852349037	Elvers (7)
29	18	Splice acceptor	-	-	-	Somatic	-	-
1	18	Missense	c.T2066C	p.L689P	Deleterious	Somatic	-	-
39	19	Frameshift deletion	c.2148delC	p.F716X	-	Somatic	-	-
47	20	Synonymous	c.G2262A	p.L754=	-	Somatic	-	-
47	20	Missense	c.G2263A	p.E755K	Deleterious	Somatic	-	-
47	20	Missense	c.G2267A	p.C756Y	Deleterious	Somatic	-	-

Variants consequences are referred to ENSCAFT00845010067.1 Ensembl canonical transcript.

*POT1* mutations were not associated with any clinico-pathological variable. Likewise, prognosis was not affected by *POT1* mutational status. Indeed, neither TTP nor LSS were significantly different between dogs carrying mutated and wild-type *POT1* (*P* = 0.5 and *P* = 0.7, respectively).

### Ki-67 evaluation

**Figure 3:**
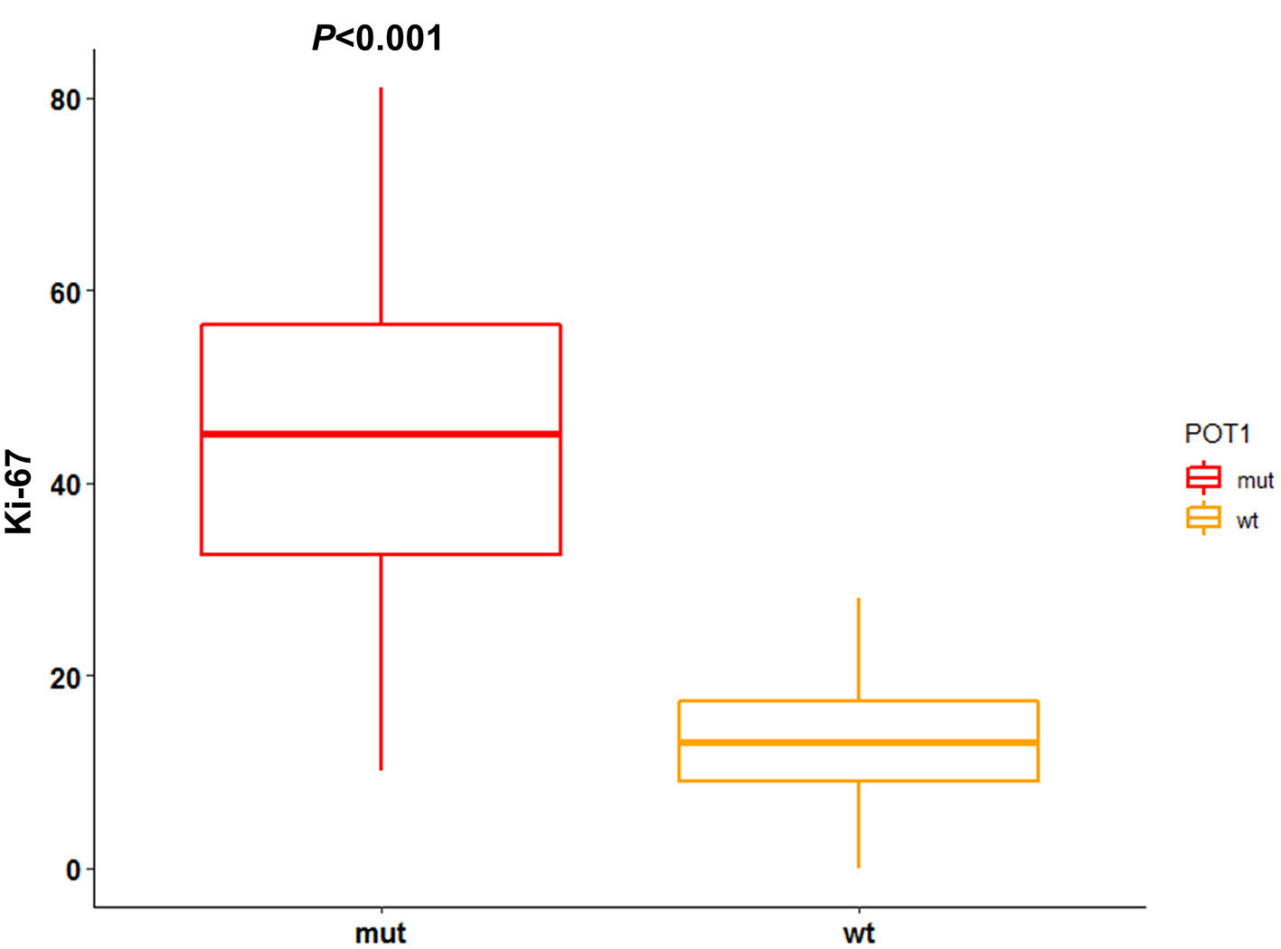
Association between *POT1* mutational status and Ki-67 index. IHC score for Ki-67 is reported as number of positive cells per 1,000 randomly selected cells excluding necrotic areas. The difference between median Ki-67 index among *POT1* mutated and wild-type dogs was assesses through Mann-Whitney test (statistical significance set at *P* < 0.05).

IHC results for Ki-67 are reported in Supplementary Table 4. Median Ki-67 index was 15.5 (range: 0–81). Ki-67 index was not associated with any clinico-pathological variable. Interestingly, median Ki-67 index was significantly higher (*P* < 0.001) in dogs with mutated *POT1* (median: 45; range: 10–81) compared to wild-type *POT1* (median: 13; range: 0–28) (Figure 3). Neither TTP nor LSS were influenced by Ki-67 IHC score (*P* = 0.7 and *P* = 0.2, respectively).

## Discussion

DLBCL represents the most common hematological malignancy in humans and dogs, and in both species this tumor is characterized by a heterogeneous clinical behavior and poor chances to anticipate the outcome (30).

In humans, the sequencing of hundreds of exomes and whole genomes from DLBCL patients allowed to identify multiple genetic subtypes characterized by specific gene mutations having prognostic significance (31–33). In dogs, some prognostic classification systems based on clinical features, such as BM infiltration or substage, have been proposed (34, 35), but molecular data were never included.

In recent WES studies, some recurrently mutated genes have been identified, including *POT1* (6, 7). In the present cohort, *POT1* mutations were retrieved in 34% of cases, showing a comparable frequency to our previous report (29%) (7). On the other hand, this frequency is higher if compared to other studies (17%) in which a generic diagnosis of cBCL was made without accounting for different histotypes (6, 25). These results may suggest that *POT1* mutations are more specific for cDLBCL rather than for other B-cell lymphoma subtypes.

POT1, along with other 5 subunits (TRF1, TRF2, RAP1, TIN2 and TPP1), constitutes the shelterin complex. This complex associates with arrays of TTAGGG repeats specific of mammalian telomeres, thus protecting chromosome ends and regulating telomerase access to 3′ ssDNA overhangs (8). Human POT1 presents two oligonucleotide/oligosaccharide binding (OB) folds (OB1 and OB2) through which it directly binds telomeric ssDNA (9, 10), and a C-terminal domain that allows POT1 heterodimerization with TPP1, which in turns anchors the remaining shelterin proteins. This interaction is necessary for POT1 localization to telomeres (36, 37). These domains are evolutionarily conserved between humans and dogs.

In the present study, 11 dogs (58% of all mutated cases) harbored a mutation within OB1_like or OB2_like domains. Among these, 10/11 (91%) were missense mutations predicted as deleterious (Figure 1, Table 1). Three of these mutations (p.P182L, p.G187E and p.R421Q) corresponded to the human p.P35L, p.G40E and p.R273Q variants that are reported in the Catalog Of Somatic Mutations In Cancer (COSMIC). Missense mutations affecting POT1 ssDNA binding domain previously identified in human studies have been functionally characterized in human and murine cells, showing that the loss of functional OB folds determines the activation of the Ataxia Telangiectasia—and Rad3—related (ATR)-mediated DDR that in turn promotes chromosome end-to-end fusions, telomere fragility and chromosomal rearrangements through the activation of the error prone non-homologous end-joining (NHEJ) mechanism (11, 12, 38). Thus, we can suppose that also in dogs these variants could reduce the affinity between POT1 and telomeric ssDNA and induce aberrant DDR at telomeres. On the other hand, a weakened affinity between POT1 and ssDNA allows telomerase to gain access to chromosome ends promoting constitutive elongation, increased telomere replication burden and telomere fragility (39, 40).

Eight dogs (42% of mutated cases) harbored a mutation within the POT1 C-terminal domain. While 10/11 (91%) of OB folds mutations retrieved in this study were missense variants, 5/8 (62%) of C-terminus variants were truncating mutations (non-sense, frameshift deletions or splice donor/acceptor variants) (Figure 1). Disruptive mutations as non-sense and frameshift variants can lead to the appearance of a premature stop codon and then to reduced expression of POT1 by means of non-sense mediated decay (41) or, alternatively, to the loss of TPP1 binding with reduced localization to telomeres. The effect of splicing variants may vary depending on the ability of the resulting protein versions to bind ssDNA or TPP1 and consequently to cause telomere elongation (42, 43).

Seven dogs harbored four different previously unreported 5′UTR variants of germline origin. Given the uncertain significance of such variants, these dogs were not included within the mutated cases group. Even if it is likely that these variants represent benign polymorphisms, we can not exclude they may have a role in cancer susceptibility. Several studies demonstrated that 5′UTR variants are able to affect proper gene expression through different mechanisms, including alternative splicing, instability of the transcript or reduced translational efficiency (44–47). To clarify the role of these variants in cDLBCL, it would be useful to extend the analysis to a control group of healthy dogs to assess their frequency within the normal population.

In a previous study, we reported reduced LSS in dogs harboring *POT1* mutations that had been treated with chemo-immunotherapy (7). However, this association was not confirmed in the present cohort. This can be explained by the fact that gene mutations are usually characterized by both incomplete penetrance and variable expressivity that, in turn, are influenced by the complex interplay between several genetic and non-genetic factors. Then, we can hypothesize that *POT1* mutations in cDLBCL may represent low penetrance variants whose pathogenic effect depends in part on the presence of predisposing genetic background.

Telomere dysfunction represents one of the key events in driving tumorigenesis (48). Among the proposed mechanisms through which *POT1* mutations may exert their oncogenic role, there is increased telomerase activity leading to telomere elongation and, in consequence, to a greater number of cell divisions that finally culminate in genomic instability and accumulation of mutations in tumor cells (49). For such reason, we evaluated Ki-67 expression through IHC in tumor tissues. Ki-67 is a proliferation marker widely used in routine diagnostic pathology and its prognostic role has been reported both in human and canine cancers (50–54). Remarkably, we retrieved a strong association between the presence of *POT1* mutations and increased Ki-67 index. To our knowledge, this is the first time that such association is reported in human or canine neoplasia. However, we did not find any association between Ki-67 index and either TPP or LSS. These results disagree with previous data reported by Sierra Matiz et al. (54) that identified an association between Ki-67 index and survival time. One possible explanation is related to the different method that was used to assess Ki-67 index selecting only areas with the highest number of positive cells rather than random spots in the lymph node. Moreover, the dogs were treated with a standard CHOP-based chemotherapeutic protocol whereas here a chemo-immunotherapy protocol consisting of CHOP + APAVAC vaccine was applied probably masking a possible biological effect (7, 27).

In conclusion, even if *POT1* mutations do not seem to have a direct impact on prognosis, we can speculate they may have a role in promoting malignant transformation through increased cell proliferation. Future studies to assess telomerase activity in these mutants and the presence of a higher grade of genomic rearrangements will be required to further elucidate the pathogenic role of *POT1* in cDLBCL.

## Data availability statement

The original contributions presented in the study are included in the article/Supplementary material, further inquiries can be directed to the corresponding author.

## Ethics statement

Ethical review and approval was not required for the animal study because the study did not fall within the application areas of the Italian Legislative Decree 26/2014 which governs the protection of animals used for scientific or educational purposes. Written informed consent was obtained from the owners for the participation of their animals in this study.

## Author contributions

LA and AF: designed the study, interpreted the data, and wrote the manuscript. LMa: provided samples and clinical data and contributed to manuscript revision. AF and LL: performed the experiments. LA and LMi: reviewed IHC slides. AF: performed data visualization and statistical analyses. NR: contributed to study design and manuscript revision. All authors contributed to the article and approved the submitted version.

## Funding

The authors declare that this study received funding from Hastim. The funder was not involved in the study design, collection, analysis, interpretation of data, the writing of this article or the decision to submit it for publication.

## Conflict of interest

Author NR was employed by Hastim. The remaining authors declare that the research was conducted in the absence of any commercial or financial relationships that could be construed as a potential conflict of interest.

## Publisher's note

All claims expressed in this article are solely those of the authors and do not necessarily represent those of their affiliated organizations, or those of the publisher, the editors and the reviewers. Any product that may be evaluated in this article, or claim that may be made by its manufacturer, is not guaranteed or endorsed by the publisher.
